# A Missense *POU4F3* Variant Associated with Autosomal Dominant Midfrequency Hearing Loss Alters Subnuclear Localization and Transcriptional Capabilities

**DOI:** 10.1155/2021/5574136

**Published:** 2021-06-21

**Authors:** Dan Bai, Xudong Zhang, Yu Li, Jing Ni, Kai Lan

**Affiliations:** ^1^Department of Otolaryngology, School of Clinical Medicine, Xi'an Medical University, Xin Wang Road No. 1, Xi'an 710041, China; ^2^Department of Otolaryngology, Second Affiliated Hospital of Xi'an Medical University, Xi'an 710038, China

## Abstract

**Background:**

The pathogenic variant, POU class 4 transcription factor 3 (*POU4F3*), is reported to cause autosomal dominant nonsyndromic hearing loss (ADNSHL). Previously, we have examined a four-generation midfrequency sensorineural hearing loss (MFSNHL) family (no. 6126) and established *POU4F3* c.602T>C (p.Leu201Pro) as a potential disease-causing variant.

**Objectives:**

We explored the structural and functional alterations that the c.602T>C (p.Leu201Pro) variant enforces on the POU4F3 protein.

**Methods:**

We utilized wild-type (WT) and mutant (MUT) *POU4F3* c.602T>C plasmid incorporation into HeLa cells to assess functional changes, by immunofluorescence and luciferase assays. To predict protein structural alterations in the MUT versus WT POU4F3, we also generated 3D structures to compare both types of POU4F3 proteins.

**Results:**

The WT POU4F3 is ubiquitously present in the nucleus, whereas the MUT form of POU4F3 exhibits a more restricted nuclear presence. This finding is different from other publications, which report a cytoplasmic localization of the MUT POU4F3. We also demonstrated that, as opposed to WT POU4F3, the MUT POU4F3 had 40% reduced luciferase activity.

**Conclusions:**

The reduced nuclear presence, combined with reduced transcriptional activity, suggests that the *POU4F3* c.602T>C variant alters cellular activity and may contribute to the pathogenicity of *POU4F3*-related hearing loss. It, also, provides more evidence of the pathophysiological characteristics of MFSNHL.

## 1. Introduction

Midfrequency sensorineural hearing loss (MFSNHL) is a severe condition that can affect hearing and speech at an early stage. Thus far, 7 genes have been identified in relation to this condition: *TECTA*, *OTOA*, *EYA4*, *COL11A2*, *CCDC50*, *POU4F3*, and *SLC44A4* [1–7]. Mutational analysis of these genes can be used in a clinical setting to aid in the molecular diagnosis of MFSNHL.

However, not much is known about the mechanism of specificity that results in MFSNHL. In our preliminary studies, we discovered the *POU4F3* heterozygous variant c.602T>C (p.Leu201Pro) as a potential molecular regulator of autosomal dominant MFSNHL in one Chinese family [8]. In this study, we examined the effects of the *POU4F3* heterozygous variant c.602T>C (p.Leu201Pro) in terms of protein localization and function, using wild-type (WT) and mutant (MUT) POU4F3 EGFP expression vectors. Previous studies on the MUT POU4F3 found them to be localized in the cytoplasm while the WT POU4F3 is localized to the nucleus [9, 10]. However, in this study, we report nuclear localization of both WT and MUT POU4F3 protein, with some spotty, inhomogeneous MUT POU4F3, but not WT POU4F3, presence near the subnuclear region. Lastly, we also showed reduced activity of the MUT POU4F3, as evidenced by luciferase activity.

## 2. Materials and Methods

### 2.1. In-Fusion Cloning and Plasmid Construction

Total RNA was extracted from HeLa cells by Trizol-based methods, and the first-strand cDNA containing human *POU4F3* (NM_002700.2) was synthesized by reverse transcription, using Oligo-dT primer. *POU4F3* expression was analyzed with PCR, using primers that overlap with PEGFP-C1. The sequences used are as follows: forward, 5′-CTCAGATCTCGAGCTCAAGCTatgatggccatgaactccaagcagcct-3′, and reverse, 5′-CGACTGCAGAATTCGAAGCTtcagtggacagccgaatacttcattcgtttct-3′. Next, *POU4F3* was cloned into a eukaryotic EGFP expression vector using the in-fusion method. The SnapGene software was used for fragment fusion and primer design. Following PCR amplification, plasmid vector linearization, and recombination, the Sanger sequencing was used to verify the insertion of *POU4F3* into the EGFP expression vector. This was used in further experimentation as the wild-type (WT) POU4F3 expression plasmid.

To construct the mutant (MUT) form of *POU4F3*, the forward primer 5′-cgcatcaagctgggggtgacccaggcggacg-3′ and reverse primer 5′-ggtcacccccagcttgatgcgccgctgctt-3′ were employed to introduce the c.602T>C missense variant to the template pEGFP-*POU4F3* via PCR. Next, Muta-Direct™ Enzyme (SBS, Beijing, China) was added to digest unmutated PCR product transcripts before subcloning the MUT *POU4F3* into an EGFP expression vector. The Muta-Direct™ Site-Directed Mutagenesis Kit (SBS, Beijing, China) was used for this construction.

### 2.2. Cell Culture and Immunocytochemistry

HeLa cells were cultured in Dulbecco's modified Eagle's medium (DMEM, Thermo, USA) with 10% fetal bovine serum (FBS, Thermo, USA) and antibiotics (Thermo, USA). For experimentation, HeLa cells were plated in cell culture plates and allowed to grow for 24 h before transfection with the indicated plasmids. After another 24 h of incubation, the cells were harvested and analyzed for immunofluorescence or luciferase activity.

For immunocytochemistry investigation, HeLa cells were plated on glass coverslips and exogenously incorporated with 1 *μ*g WT or MUT POU4F3 using the PEI transfection reagent, following the manufacturer's guidelines. Lastly, the expression was assessed using an inverted fluorescence microscope of the DragonFly confocal imaging system (Andor, Oxford, England).

### 2.3. Luciferase Assay

The Prox1 luciferase reporter plasmid was constructed using primers 5′-CTAGCAAAATAGGCTGTCCC-3′. HeLa cells were plated in 24-well plates and allowed to grow till 70% confluency, before exogenous incorporation of the following plasmids into the cells: WT or MUT EGFP-*POU4F3* (0.5 *μ*g), firefly (PGL3-Basic-Prox1) or empty vector (PGL3-Basic) (0.5 *μ*g), and Renilla (TK) reporter plasmid (0.01 *μ*g). Luciferase activity was assessed using a Promega E2920 kit (Promega, USA) 48 h after cotransfection. The results were based on the average of 3 separate experiments.

### 2.4. Model Building and Structural-Based Analysis

The three-dimensional (3D) modeling of the human WT and p.Leu201Pro MUT was conducted using SWISS-MODEL, a homology modeling software available at http://swissmodel.expasy.org/workspace/. This approach used the complete protein sequence data available at the NCBI GenBank (NP_002691.1). Data from the homology models were visualized using Swiss-PdbViewer 4.1.

## 3. Results

### 3.1. Mutant POU4F3 Alters the Subnuclear Localization of POU4F3 Protein

**Figure 1:**
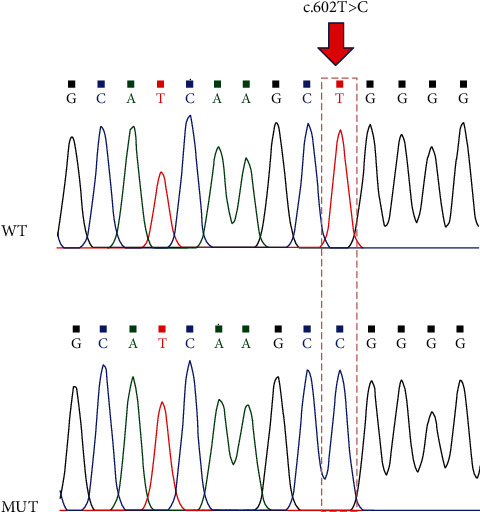
Sanger sequencing of wild-type (WT) and mutant (MUT) POU4F3. Red arrow indicates the position of the c.602T>C variant in *POU4F3*.

POU4F3 acts as a transcription factor that interacts with and modulates the expression of target-specific genes [11, 12]. Therefore, to elucidate the function of POU4F3, we first introduced a c.602T>C variant in the human *POU4F3* gene and confirmed the variant using Sanger sequencing, as illustrated in Figure 1.

**Figure 2:**
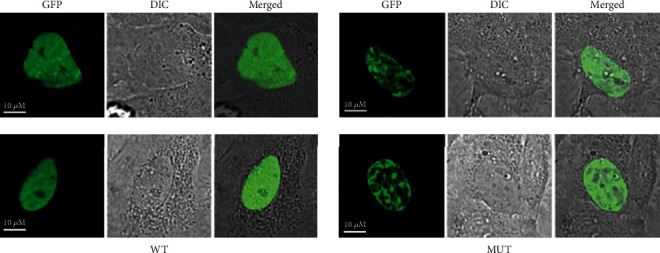
Representative fluoroscopy images depicting subcellular localization of wild-type (WT) and mutant (MUT) POU4F3. Transient expression of WT and MUT POU4F3 in HeLa cells showing nuclear localization of both WT and MUT POU4F3. Compared to WT POU4F3, the MUT POU4F3 exhibited a region-specific localization within the nucleus. GFP: green fluorescent protein; DIC: differential interference contrast microscope.

The WT and MUT human *POU4F3* were then cloned into an EGFP expression vector and incorporated into HeLa cells for the study of protein subcellular localization. We demonstrated that both WT and MUT POU4F3 exclusively localized in the nucleus (Figure 2). However, the level of expression was vastly different. The WT POU4F3 was equally expressed all over the nucleus, whereas the MUT POU4F3 had a patchy inhomogeneous expression within the nucleus and appeared to be localized in certain regions of the nucleus. Moreover, the physiological effects of this redistribution of MUT POU4F3 were unclear.

### 3.2. The c.602T>C Variant Reduced the Transcriptional Capability of POU4F3

**Figure 3:**
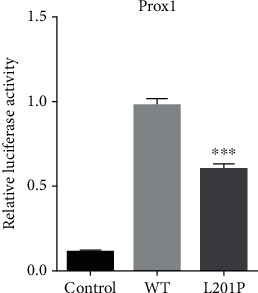
Transcriptional capability of the wild-type (WT) versus mutant (MUT) POU4F3 protein. Relative luciferase activity was measured after HeLa cellular incorporation with the Prox1 firefly luciferase reporter or empty control, Renilla control, and either WT or MUT POU4F3 expression vector for 24 h. In this model, POU4F3 was able to bind to the POU4F3 recognition sequence within the Prox1 firefly luciferase reporter to activate luciferase expression, measured with a dual-luciferase detection kit E2920 (Promega, USA) and full-wavelength microplate reader (Thermo Fisher, USA). The presented data is an average ± standard deviation (SD) of 3 separate experiments with 3 replicas/group in each experiment.

To assess the ability of WT and MUT POU4F3 to modulate target-specific gene expression, we cloned a *POU4F3* target gene *Prox1* into a luciferase reporter plasmid, regulated by an upstream *POU4F3* recognition element RVP3. Next, we incorporated into HeLa cells the constructed *Prox1* firefly luciferase reporter or empty vector, Renilla control, and either WT or MUT *POU4F3* expression vector for 24 h. As shown in Figure 3, WT POU4F3 exhibited a 10-fold increase in luciferase activity, relative to the empty vector. Comparatively, the MUT POU4F3 elicited a 6-fold increase in luciferase activity, as opposed to the negative control.

### 3.3. Structural Modeling of p.Leu201Pro

**Figure 4:**
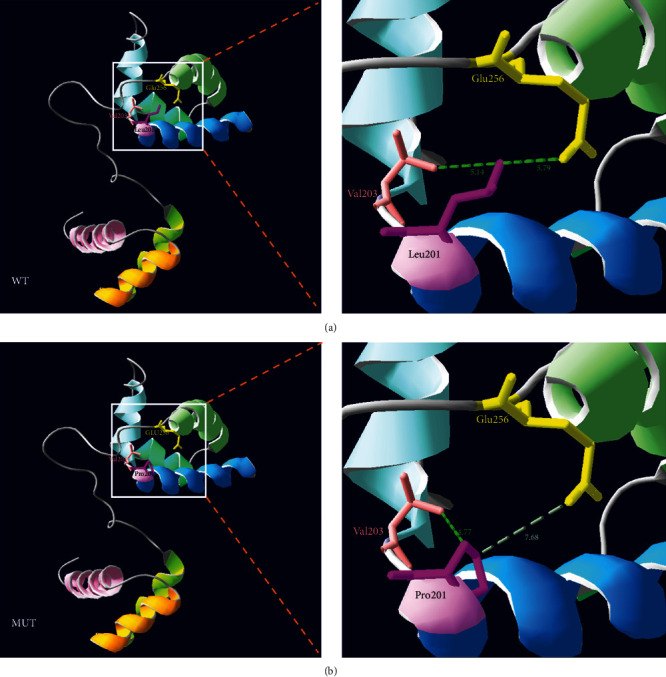
Evaluation of the transcriptional capabilities of wild-type (WT) and mutant (MUT) POU4F3 protein in its modulation of Prox1. 3D structural comparison of WT and MUT POU4F3 protein. (a) WT POU4F3 contains leu201 at the end of the first *α*-helix of the POU-specific domain. The distance between the side chains linking Leu201 to Glu256 and Leu201 to Val203 (dotted lines) is shown. (b) MUT POU4F3, with a Leu201 to Pro201 alteration, exhibited smaller side chains and different H-bond distances.

The SWISS-MODEL software was employed to generate two p.Leu201Pro molecular models using the Prox1 binding sequence of WT and MUT POU4F3 protein, specifically residues 185–331, and the data used was based on the crystalline structure of the POU/HMG/DNA ternary complex (PDB ID: 1gt0.1). According to our model, a 50% sequence identity existed between WT POU4F3 and its target template. Using Swiss-PdbViewer 4.1.0, we also demonstrated that the WT POU4F3 protein has two long side chains and the distances between side chains Leu201 and Glu256 and Leu201 and Val203 were 5.79 and 5.14, respectively (in Figure 4(a)). The MUT POU4F3, however, contained the amino acid Pro201, in place of Leu201, which markedly altered the distances between the side chains which are 7.68 and 4.77 (Figure 4(b)). These predictions may explain the reduced transcriptional activity seen with the MUT POU4F3, relative to WT POU4F3.

## 4. Discussion

The pathogenesis of MFSNHL has a strong genetic component. Until now, 7 genes have been recognized as responsible for this debilitating condition: namely, *TECTA*, *EYA4*, *OTOA*, *COL11A2*, *CCDC50*, *SLC44A4*, and *POU4F3*. Among these seven genes, variants in *TECTA* are most frequently reported. *TECTA* encodes *α*-tectorin, the major component of noncollagenous glycoprotein of the tectorial membrane, and has a role in intracochlear sound transmission. It had been suggested in a previous report that *TECTA*-related MFSNHL appeared to be associated with the position of the variants in the ZP domain of *α*-tectorin. This domain is responsible for secretion and polymerization of extracellular proteins into supramolecular structure. An in vitro study showed differences of localization patterns between wild-type and mutant TECTA and suggested that mutant TECTA may lead to a lack of assembly of secretion and may reduce the incorporation of *α*-tectorin into the tectorial membrane [13]. *OTOA* encodes otoancorin, a protein that acts as a glycosylphosphatidylinositol (GPI) anchorage, and is important for limbal attachment of the tectorial membrane, which is important for conditioning proper stimulation of the inner hair cells [14, 15]. The similarities between the clinical characteristics of hearing loss in patients with *OTOA* and *TECTA* disease-causing variants reflect the similar mechanism of hearing loss caused by tectorial membrane impairment [3]. However, the detailed underlying mechanism associated with MFSNHL remains unknown.

The POU4F3 protein contains 338 amino acids and is encoded by 2 exons. It belongs to the POU domain family of transcription factors (TFs) and is expressed particularly in the inner ear hair cells. This TF plays a major role in the maturation, differentiation, and maintenance of inner ear hair cells [9–11]. The first *POU4F3* variant was first discovered by Vahava et al. in 1998 in a Jewish family suffering from progressive hearing loss. All deaf members of this family carried an 8-base deletion in exon 2 of the *POU4F3* gene. This variant interrupts its own translation, after the first *α*-helix, resulting in an incomplete protein with restricted transcriptional control [7, 16].

Thus far, 32 variations of the *POU4F3* gene have been associated with ADNSHL, which presents with a vast range in age of onset and disease severity among different ethnic populations [7, 12, 17–24]. In a clinical audiological analysis of 15 ADNSHL families associated with *POU4F3* pathogenic variants, it was shown that 20% of the patients had early MFNSHL, which progressed to high-frequency hearing loss and further led to complete hearing loss. Moreover, it was reported that patients with truncated *POU4F3* variants exhibited earlier onset, but slow progression of hearing loss, relative to patients with nontruncated variants [12]. Using next-generation sequencing, another study demonstrated that the *POU4F3* variant makes up a considerable portion (3/18) of the ADNSHL population among the Han Chinese [22]. Our previous study identified *POU4F3* c.602T>C (p.Leu201Pro) as a pathogenic variant of MFSNHL in one Chinese family [8]. Owing to the change from Leu201 to Pro201 in the *POU4F3* variant, it is possible that the mutated TF binds to target-specific DNA with less specificity and therefore results in reduced target gene expression. To test this, the transcriptional activities of WT and MUT POU4F3 were assessed in HeLa cells using a previously described Prox1 luciferase reporter construct [25]. As expected, the MUT POU4F3 was shown to have reduced transcriptional activity, as opposed to WT POU4F3.

We also constructed a 3D molecular model of WT and MUT forms of the POU4F3 Prox1 binding site and conducted subsequent functional assays to determine the role of the c.602T>C variant of *POU4F3* in regulating Prox1. As depicted in the 3D model, a switch from Leu201 to Pro201 in the MUT POU4F3 resulted in the long chain of leucine to be replaced by a pyrrolidine ring of proline. This structural alteration disrupted the normal tertiary structure of the DNA-binding domain of POU4F3, thereby reducing POU4F3-DNA interaction and transcriptional control. In subcellular assays, the MUT POU4F3 was found to be uniquely localized to certain regions of the nucleus. This unique behavior of MUT POU4F3 was not reported before. It is our hypothesis that the structural and functional changes brought on by this missense variant of c.602T>C result in MFNSHL, the condition observed in patients of the family presented in our previous study [8].

In summary, we performed structural and functional analyses on a previously identified pathogenic variant c.602T>C (p.Leu201Pro) of *POU4F3* to elucidate its role in MFNSHL. We demonstrated that this variant altered the protein such that its binding to target-specific DNA became more restrictive and its transcriptional ability was reduced. The conclusions of this paper will add insight into the growing knowledge of information on the pathogenesis of *POU4F3*-associated hearing loss.

## Data Availability

The data used to support the findings of this study are available from the corresponding author upon request.
